# Effects of health intervention programs and arsenic exposure on child mortality from acute lower respiratory infections in rural Bangladesh

**DOI:** 10.1186/s12942-016-0061-9

**Published:** 2016-09-01

**Authors:** Warren C. Jochem, Abdur Razzaque, Elisabeth Dowling Root

**Affiliations:** 1 Department of Geography and Environment, University of Southampton, University Road, Southampton, SO17 1BJ UK; 2 Health Systems and Population Studies Division, icddr,b, 68, Shaheed Tajuddin Ahmed Sarani, Mohakhali, Dhaka, 1212 Bangladesh; 3 Department of Geography, Division of Epidemiology, The Ohio State University, 1036 Derby Hall, 154 North Oval Mall, Columbus, OH 43212 USA

**Keywords:** Acute lower respiratory infection, Arsenic, Child mortality, Spatial scan statistic, Zero-inflated negative binomial, Bangladesh

## Abstract

**Background:**

Respiratory infections continue to be a public health threat, particularly to young children in developing countries. Understanding the geographic patterns of diseases and the role of potential risk factors can help improve future mitigation efforts. Toward this goal, this paper applies a spatial scan statistic combined with a zero-inflated negative-binomial regression to re-examine the impacts of a community-based treatment program on the geographic patterns of acute lower respiratory infection (ALRI) mortality in an area of rural Bangladesh. Exposure to arsenic-contaminated drinking water is also a serious threat to the health of children in this area, and the variation in exposure to arsenic must be considered when evaluating the health interventions.

**Methods:**

ALRI mortality data were obtained for children under 2 years old from 1989 to 1996 in the Matlab Health and Demographic Surveillance System. This study period covers the years immediately following the implementation of an ALRI control program. A zero-inflated negative binomial (ZINB) regression model was first used to simultaneously estimate mortality rates and the likelihood of no deaths in groups of related households while controlling for socioeconomic status, potential arsenic exposure, and access to care. Next a spatial scan statistic was used to assess the location and magnitude of clusters of ALRI mortality. The ZINB model was used to adjust the scan statistic for multiple social and environmental risk factors.

**Results:**

The results of the ZINB models and spatial scan statistic suggest that the ALRI control program was successful in reducing child mortality in the study area. Exposure to arsenic-contaminated drinking water was not associated with increased mortality. Higher socioeconomic status also significantly reduced mortality rates, even among households who were in the treatment program area.

**Conclusion:**

Community-based ALRI interventions can be effective at reducing child mortality, though socioeconomic factors may continue to influence mortality patterns. The combination of spatial and non-spatial methods used in this paper has not been applied previously in the literature, and this study demonstrates the importance of such approaches for evaluating and improving public health intervention programs.

## Background

Acute lower respiratory infections (ALRI) are the leading cause of childhood morbidity and mortality globally and are responsible for 18 % of all deaths in children under 5 years [1, 2]. Children in less-developed countries bear the majority of the burden of disease, experiencing 97 % of the estimated 156 million new cases of pneumonia each year [3–6]. Regionally, South and Southeast Asia have some of the highest rates of respiratory infection-related mortality, with approximately 21 % of all deaths in children under 5 years old attributed to pneumonia [1]. In Bangladesh ALRI are a leading cause of morbidity and mortality among children [4, 7, 8]. Children on average experience between 0.23 and 0.47 events per year [5, 6] and over 25,000 die from pneumonia alone each year [1]. Prior studies have found that childhood ALRI is associated with poverty, malnutrition, indoor air pollution, crowded living conditions, as well as access to medical care [2, 3, 7, 9], all factors which affect immune status or increase exposure to pathogens or lung irritants [3, 10].

ALRI continues to be a serious health concern, though it is largely treatable and preventable. Vaccines designed to target two of the major causes of ALRI, pneumococcus (*Streptococcus pneumonia*) and Hib (*Haemophilus influenzae* type b), are currently available and have been found to be effective against invasive disease; however, there are significant political, economic, and logistical challenges to distributing vaccines, particularly in developing countries [11]. Additionally, since a wide variety of pathogens can cause ALRI, vaccines can only prevent a small proportion of disease and a significant number of new cases are still likely to develop [5, 10]. In the absence of these prevention strategies, community-based intervention programs designed for early ALRI case detection and treatment with antibiotics can be successful in reducing child mortality from ALRI [12].

In Matlab, Bangladesh an ALRI intervention program implemented in 1988 was successful in quickly reducing child mortality by over 50 % [13]. An evaluation of the program by Ali and colleagues [7] identified considerable geographic variation in the ALRI mortality rates experienced by groups of households (known as *baris*) in that community; however, their work was largely a descriptive visualization of smoothed rates to accompany a non-spatial regression analysis. They stopped short of testing whether those geographic patterns of mortality events occurred due to chance and exploring which contextual and environmental factors contributed to the observed differences over space. ALRI remains a persistent problem in Matlab [6, 13, 14] and there are local clusters of elevated all-cause child mortality that remain unexplained [15], necessitating this spatial study of ALRI mortality patterns.

In addition to ALRI, the widespread contamination of drinking water by inorganic arsenic in Bangladesh (see [16]) is a health threat which must be considered when examining the effects of the health intervention programs in Matlab. The arsenic contamination is an unintended consequence of successful programs begun in the 1970s that installed wells, also called *tubewells*, across the region in order to provide clean drinking water and prevent diarrheal diseases [17, 18]. Arsenic-contaminated water has no distinguishing color, smell, or taste, and it was not routinely tested for in well installations, so the contamination was not detected until health problems were identified in the mid-1990s.

Inorganic arsenic is a potent toxin that causes wide-ranging health problems as a result of its damaging effects on the immune system [19, 20]. Despite being ingested rather than inhaled, lung cells seem particularly sensitive to arsenic-contaminated drinking water as the arsenic disrupts the inflammatory response and innate immune system signaling, increasing the risk of a lower respiratory infection [21, 22]. Previous epidemiological studies have found that people exposed to arsenic are more likely to report symptoms such as frequent coughs and to show decreased lung function [23–28]. Exposure to arsenic is also associated with increasing mortality from lung cancer [29], bronchiectasis [30], and tuberculosis [31], as well as decreasing lung function [32], and increasing susceptibility to lower respiratory infections [33, 34].

The objectives of this study are twofold. The first objective is to further develop spatial statistical methods to identify local spatial clusters while testing for known and hypothesized risk factors. This work makes use of zero-inflated negative binomial regression models (ZINB) [35] and the spatial scan statistic [36], and it demonstrates the potential for using non-spatial regression techniques to adjust spatial cluster detection tests for known risk factors. The second objective is to apply these methods to re-examine an ALRI control program that was introduced in Matlab, Bangladesh and to evaluate its effects on local child mortality patterns. While the ALRI intervention program was discussed by Ali et al. [7], the methodological approach developed here provides a more appropriate test of the geographic patterns of ALRI mortality in young children. Studying spatial disease patterns is vital for understanding the role of known risk factors in order to provide more targeted and appropriate public health interventions. Spatial analysis also can be hypothesis generating and exploratory of additional factors that could generate disparities in mortality. Arsenic exposure was not considered in any earlier study evaluating the impact of the ALRI program in Matlab and the possible link between child mortality from ALRI and arsenic exposure has not been well studied. Therefore this paper reports on the population-level relationship between arsenic in drinking water and child mortality from ALRI.

## Methods

### Study area

**Fig. 1:**
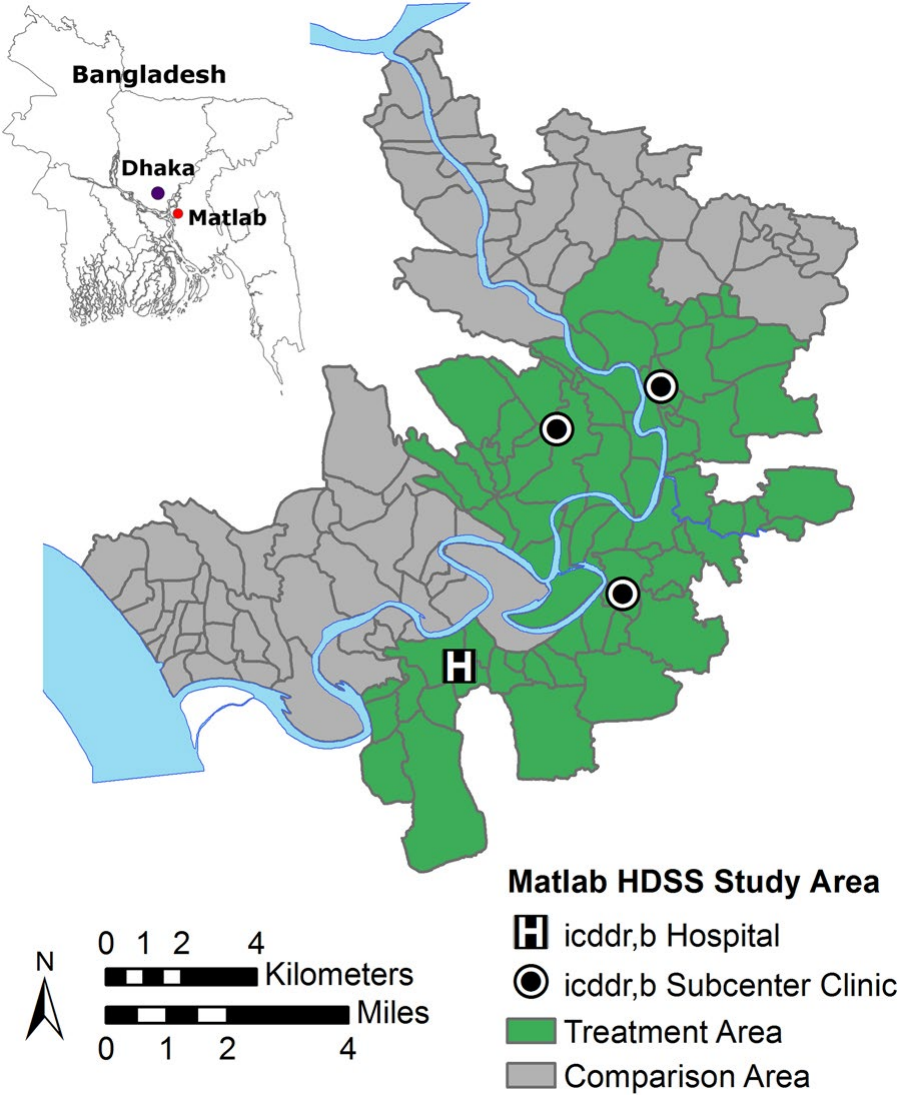
The study area of Matlab, Bangladesh. The Health and Demographic Surveillance System (HDSS) area of Matlab is located in central Bangladesh. Villages were divided into two groups, one group had access to specialized medical facilities operated by icddr,b and treatment for acute lower respiratory infections and the other group (referred to as the comparison group) received standard government care. Data source: icddr,b CPUCC/GIS Unit

This study was conducted in Matlab, a rural group of 142 villages located approximately 50 km southeast of the capital city Dhaka in central Bangladesh (Fig. 1). Since the 1960s, Matlab has been the site of a comprehensive health and demographic surveillance system (HDSS) organized by icddr,b (formerly known as the International Center for Diarrheal Disease Research, Bangladesh) which has recorded all births, deaths, and migrations as well as conducted periodic censuses in the region. The population of Matlab lives primarily in patrilineally-related groups of housing units known as *baris*, which are the unit of analysis for this study.

Starting in 1977 icddr,b initiated a series of community-based projects in Matlab providing family planning services, immunizations, and perinatal care [37]. A key feature of the project was the use of local, specially-trained, female community health workers (CHW) for service delivery and demographic surveillance. Referred to collectively as the maternal and child health and family planning program (MCH/FP), the interventions in Matlab were successful in reducing fertility and mortality by increasing the prevalence of contraceptives and vaccine use [37–39]. The MCH/FP interventions were implemented only in half of the villages in Matlab, called the “treatment area” (Fig. 1). The other villages in Matlab form two “comparison areas” adjacent to the treatment area, one in the north and the other in the southwest (Fig. 1). These areas received standard government services, but both the treatment and comparison area populations are recorded in the HDSS. Prior to the start of the MCH/FP the treatment and comparison areas had similar health and demographic measures.

### ALRI control program

Beginning in 1988 the MCH/FP project expanded to include an ALRI control program [13, 40]. This community-based program was designed to reduce mortality in children under 5 years old from ALRI through a combination of health education activities for caregivers, early case detection, and management of cases using antibiotic treatment. Three outpatient, subcenter clinics as well as hospital facilities were used to treat ALRI (locations shown in Fig. 1). The ALRI program was managed by the CHWs who regularly visited households to give caretakers information on symptoms and treatment of ALRI and to identify new ALRI cases using a modified WHO case definition based on respiratory rates and other visible symptoms such as chest retractions [40, 41]. ALRI was classified as mild, moderate, or severe. Mild cases were monitored by CHW and mothers were given additional health information for supportive care. A moderate case, diagnosed as respiratory rates greater than 50 breathes per minute but without other symptoms, was treated with antibiotics. Severe pneumonia cases, defined by the presence of chest retractions and other symptoms along with respiratory rates above 50 breathes per minute or any lung infection in children under 1 month old regardless of symptoms, were referred to the icddr,b hospital in Matlab where oxygen, intravenous fluids and antibiotics were available.

### Arsenic exposure

While contamination of drinking water by arsenic occurs throughout Bangladesh, the area of Matlab experiences some of the highest rates as a result of local geologic conditions [42]. The first arsenic survey of wells within Matlab conducted in 2002–2003 found 62 % of over 13,000 wells had arsenic levels above the Bangladeshi-government recommended level of 50 µg/L [43]. Studies using the HDSS data from Matlab have found that exposure to arsenic-contaminated drinking water increases risks of skin lesions, hypertension, diabetes mellitus, lung disease, and is resulting in excess adult and infant mortality [44]. Even within Matlab, arsenic contamination exhibits local-scale spatial variation due to differences in geologic conditions as well as the depth of the well [45]. Wells that tap shallower, younger aquifers are more likely to be contaminated with arsenic, while deeper wells beyond 150 m are almost entirely free of arsenic [42]. Therefore, even closely neighboring households can have different levels of arsenic exposure, and this variation in arsenic could contribute to spatial variation in disease risk.

### Study data

Data for this study come from the Matlab HDSS records and include all deaths reported from pneumonia or other ALRI. Similar to Ali et al. [7], this study focuses on the population with the highest rates of ALRI, children under 2 years old, and during the years immediately following the full implementation of the ALRI control program (1989–1996) to evaluate the program’s effects. This period also covers the years before knowledge of arsenic contamination was widespread and the use of wells for drinking water was common, resulting in high levels of exposure [46, 47]. HDSS data for each individual in Matlab can be linked across study years to incorporate additional census and survey data as well as linked spatially to the *bari* locations in the Matlab Geographic Information System (MGIS) [48]. Person-years for children under age 2 years in each *bari* define the population at risk. To calculate person years, each child is linked to their *baris* using a unique identification number and then followed through the HDSS records until one of three outcomes occurs: permanently out-migrate, die, or turn 2 years old. HDSS events are aggregated by *bari* across the study period to calculate the total ALRI deaths and person-years of children under 2 years (the population at risk). *Baris* are represented as point locations in the MGIS and become the unit of analysis for all analyses. A total of 7846 unique *baris* were identified during the 1989–1996 study period; though 1157 did not contain any children and were excluded from the analyses because they have no population at risk, leaving 6693 *baris*.

A *bari*-level estimate of arsenic exposure was created using data from the Matlab arsenic survey conducted in 2002–2003 which measured arsenic by lab-based hydride generation atomic absorption spectrometry (HG-AAS) and mapped wells using global position system receivers [49]. A retrospective estimate of potential exposure was created in two steps. First the 1996 census was used to find households drinking from tubewells versus surface water sources (i.e. ponds, rivers, canals). Surface water is largely free of arsenic, but *baris* with households using tubewells are potentially exposed. *Baris* were assumed to experience the average concentration of arsenic in all wells owned by the *bari*. Following a similar procedure to Carrel et al. [50], well ownership was based on HDSS identification numbers linking wells to *baris*. If a *bari* did not have a tubewell defined by the identification number, but still reported drinking from a well, the arsenic concentration of the nearest well (based on straight line distance) identified in the MGIS was used. Arsenic levels are generally stable over time [42, 51, 52], lending support for this approach; however, the differences in study years and arsenic measures presents certain limitations to our study which we discuss later.

Additional covariates used when adjusting the models include whether the *bari* was located in the treatment area, *bari*-level socioeconomic status, and cost distance to an ALRI treatment center. Area-level socioeconomic status (SES) has been shown to influence lower respiratory infections in other contexts [53, 54], and, within Matlab, SES is linked with nutritional status which has implications for a child’s immune system health [40]. It can be difficult to estimate household wealth or status in contexts without well-defined income. Therefore, similar to previous studies [15, 55–57], SES was estimated using a principal component analysis (PCA) of 1996 census data indicating the ownership of household assets (blanket/quilt, bed, lamp, watch, bicycle) as well as the material of the house walls. The *bari*-level SES score is the average of the first principal component score for all households in the *bari*. The *bari* SES was then divided into quintiles, and dichotomized with the top two quintiles considered high socioeconomic status.

Cost distance is a measure of accessibility or effort to reach a treatment center that considers both distance and physical barriers. A similar measure was found to be significant in the earlier study of ALRI mortality in Matlab [7] and the same procedure is used here. The accessibility from each *bari* point location was measured as the minimum number of cells on a raster surface (30 m resolution) needed to reach the nearest ALRI treatment center. Crossing one cell on land is assigned a value of 1, while travelling across water incurs a cost value of 5 per cell, reflecting a greater travel effort needed. Roads were not taken into consideration for accessibility because they were not well-developed during the study period. Calculations were performed in ArcGIS 10.2.1 (ESRI, Redlands, CA USA). Cost distance is included in the models for all *baris* even though all clinics are located in the treatment area in order to be consistent with the previous study [7]. An additional consideration and justification for including the measure is the possibility of spatial spillover effects—households in the comparison area who are closer to treatment area clinics, could have their health seeking behavior influenced by their neighbors.

Higher densities of people can increase the transmission of pathogens that cause ALRI [58]. The population density of the area surrounding each *bari* was calculated using a circular neighborhood with a radius of 200 meters centered on each *bari*. This distance was selected to replicate the measure used by [7]. The total population of each *bari* in the 1993 census was used for the density calculation.

### Statistical methods

The analyses proceeded in two stages. First, zero-inflated negative binomial (ZINB) regression was used to model *bari*-level mortality rates. ZINB models are two-part mixture models that adjust for overdispersion (when the variance exceeds the mean) in the outcome and excess zeros produced by rare events [35, 59]. The results tables show both components of the model separately, though they are estimated at the same time. The first component is the count model using the negative binomial distribution and log-link function used to describe the number of ALRI mortality events at a *bari*. The parameter estimates for this component describe the associations with counts of mortality events after accounting for excess zeros. The negative binomial distribution includes zero as a valid event, but for rare events, the inflated number of zeroes can bias the model. The second component explicitly models the likelihood of zero mortality events (relative to 1 or more) at a *bari* using a binary model. For our models, both components used the same covariate adjustments. The log of the person-years of children under 2 years was included as an offset population to account for differences in the population at risk across *baris*. Covariates were added iteratively and goodness of fit was assessed with deviance and Akaike Information Criterion (AIC) scores. All analyses were conducted using R 2.12.1 [60]. ZINB regression was carried out with the *zeroinfl* procedure in the *pscl* package [61, 62]. Only the results of the final models are presented here.

Following the regression models, spatial cluster detection tests were performed using the spatial scan statistic implemented in the SaTScan software package [36, 63]. The spatial scan statistic is a technique for detecting local clusters which operates by placing a large number of circles of varying radii at each location, and calculating the ratio of observed events to expected events in the population within each scanning window. A likelihood ratio test is calculated for each circle to test whether the observed to expected ratio within a scanning window is different from the risk in the total population outside of the scanning window. The maximum likelihood ratio identifies the most likely cluster at a location and statistical significance is determined using Monte Carlo simulations of the locations of observed cases under the null hypothesis that events are distributed over the study area proportionally to the population. We scanned for high or low clusters of ALRI. The number of deaths to children under 2 years in each *bari* is assumed to be Poisson distributed. The maximum scanning window was limited to up to 50 % of the population at risk. Additional tests were performed with this setting limited to ≤10 % of the population at risk in order to assess whether smaller clusters or alternative patterns were being hidden. In all tests, clusters were considered to be significantly different from the null hypothesis of complete spatial randomness at the α = 0.1 level as determined by 999 Monte Carlo simulations. Only clusters with no geographic overlap are presented.

Three separate cluster analyses were performed in this study—one unadjusted and two adjusted models. The unadjusted model used observed counts of ALRI and the person-years at risk to scan for clusters. The adjusted models first incorporated only the treatment area as a covariate. Next the fitted values from the ZINB models, which estimated ALRI mortality counts after adjusting for known risk factors (including the treatment area), were used. In the adjusted models, the observed number of deaths in a *bari* remains the same in each analysis and is derived from the HDSS records. The expected number of deaths at a location, used as the denominator in the spatial scan calculation, varies as covariate adjustments are made. These changes in the expected cases and subsequent changes in the risk within a scanning window are the basis for interpreting the relationships between risk factors and disease patterns. If a covariate is related to an increase in mortality rate, the expected number of deaths will be increased following adjustment and the observed to expected ratio will be reduced compared with a non-adjusted analysis. Thus, if a significant high cluster found in a given location in an unadjusted analysis is no longer significant after introduction of a covariate adjustment, we can say that the observed cluster was due to the uneven spatial distribution of that risk factor. Clusters which persist or appear after adjustment are not fully explained by a given model. Therefore cluster detection can be useful for generating research questions and hypotheses regarding additional risk factors for a given disease process.

This study differs from most previous applications of the spatial scan statistic by using regression models to calculate expected mortality counts. This approach is more flexible than entering covariate information into the SaTScan software, which is limited to a small number of categorical covariates. The regression preprocessing step allows the spatial scan statistic to effectively use continuous covariates as well as take advantage of more sophisticated, non-spatial modeling that could include non-linear relationships or more sophisticated model forms such as multilevel models [64].

The first cluster analysis is unadjusted to provide a baseline for comparison. Without adjustment, the expected number of deaths in a *bari* is proportional to the population of children under 2 years old at risk. As the ALRI control program was implemented in a geographically defined area of Matlab and did reduce mortality, we can be confident that it will also affect spatial mortality patterns. The second analysis introduces an adjustment for whether a *bari* is located within the treatment area. In this analysis the expected mortality events are calculated using the treatment/comparison area-specific rates. The third analysis uses expected counts calculated from the previously constructed ZINB model that adjusts for treatment area effects, as well as *bari*-level socioeconomic status, cost distance to a medical facility, and exposure to inorganic arsenic from contaminated drinking water. This model incorporates continuous measures of the cost surface and arsenic exposure, as well as the categorical variables for treatment area and high socioeconomic status. Results from all three analyses are presented graphically after importing the results from SaTScan into ArcGIS, to identify the locations of significant clusters as well as in tabular format to compare changes in likelihood and relative risk among clusters. This study (protocol number 12-0183) was reviewed by the Institutional Review Board of the University of Colorado Boulder and granted exempt status. All data were anonymized by icddr,b before being released to the investigators.

## Results

**Fig. 2:**
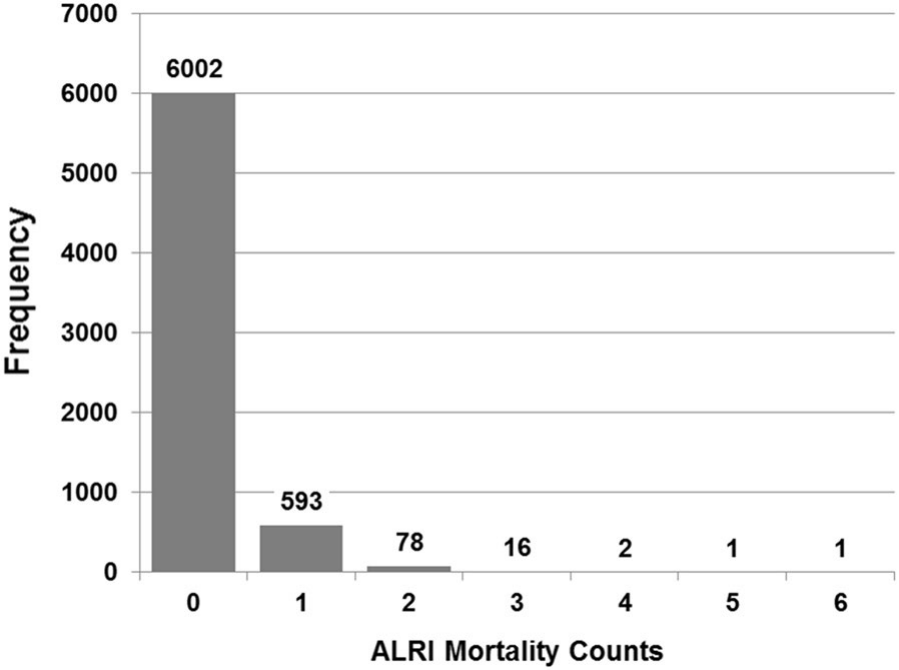
Deaths in children under 2 years old due to Acute Lower Respiratory Infections (ALRI). The distribution of child mortality from ALRI reported by group of households (known as *baris*) in Matlab, Bangladesh from 1989 to 1996 is highly zero-inflated. Data source: icddr,b Matlab Health and Demographic Surveillance System

Between 1989 and 1996, 816 deaths from ALRI were reported in children under 2 years old (276 in the treatment area, 540 in the comparison area), and the mortality rates were almost 50 % lower within the treatment area (6.18 per 1000 person-years vs. 10.74 per 1000 person-years). These deaths are 84 % of ALRI deaths across all ages in Matlab during that period. Out of 6693 *baris*, only 691 (10.3 %) reported at least 1 death, producing a larger proportion (89.7 %) of *baris* without any deaths (Fig. 2). The distribution of mortality events in the *baris* was also slightly overdispersed (variance divided by the mean = 1.278), prompting our decision to use the more flexible negative binomial rather than Poisson distribution.

Table 1 presents characteristics of the *baris* stratified by treatment and comparison area villages. Relative to the comparison area, the treatment area had slightly smaller populations at risk in each *bari*, lower average arsenic exposure levels, a larger proportion (62.9 %) of higher socioeconomic status *baris*, and a lower average population density. As all clinics are located within the treatment area, these *baris* also had lower average travel cost distances to reach a clinic. Use of tubewells and exposure to arsenic is widespread: only 179 *baris* (2.7 %) reported using a surface water source in 1996. These *baris* were assigned an arsenic exposure of 0. For the remaining *baris*, 29.4 % (n = 1912) were linked by identification number to one or more wells and assigned the average arsenic levels. The final 4781 *baris* were assumed to use the nearest tubewell, which was an average of 78 meters away from the *bari.* Overall, 69.3 % of all *baris* (n = 4636) have estimated arsenic levels above the Government of Bangladesh-recommended level of 50 μg/L.

**Table 1:** Sample characteristics

	Treatment	Comparison	*p* value
*Bari* person-years (children <2 years)			
Mean (SD)	13.7 (13.3)	14.6 (16.6)	0.018
Arsenic (µg/L)			
Mean (SD)	186.9 (183.0)	253.1 (225.3)	0.001
Higher socioeconomic status			
N (%)	2046 (62.9)	1937 (56.3)	0.001
Cost distance to clinic			
Mean (SD)	2225.4 (1161.8)	5864.8 (2224.4)	0.001
Population density (population in 1993 per sq. km)			
Mean (SD)	2500.0 (2143.9)	2812.5 (1633.9)	0.001
N	3251	3442	

Descriptive comparisons of *bari* characteristics of the treatment and comparison areas within Matlab, Bangladesh. Tests of differences based on two sample t-tests or Chi squared, as appropriate. Data source: icddr,b, Matlab Health and Demographic Surveillance System

Table 2 shows the results of the final model from the ZINB analysis. The upper panel of the table presents coefficients and standard errors from a negative binomial component while the lower panel shows coefficients and standard errors from the logit model component predicting that a *bari* reports no deaths. In preliminary tests population density was found not to be significantly related to the outcome and omitted from results shown here. While none of the covariates were significantly associated with reporting zero events, being located within the ALRI treatment area and living in a high socioeconomic status significantly reduced mortality rates. Potential arsenic exposure and the cost distance to the nearest clinic were not significantly associated with ALRI mortality in children <2 years.

**Table 2:** Regression results

	β	SE	*p*
*Negative binomial model*			
Treatment area	−0.580	0.108	0.000
Arsenic (100 µg/L)	0.009	0.020	0.644
High socioeconomic status	−0.302	0.076	0.000
Cost distance to clinic^a^	−0.092	0.052	0.077
(Intercept)	−4.359	0.073	0.000
Log (theta)	1.704	0.595	0.004
*Zero-inflated model*			
Treatment area	11.985	76.145	0.875
Arsenic (100 µg/L)	−2.633	2.769	0.342
High socioeconomic status	1.007	1.334	0.450
Cost distance to clinic^a^	1.206	1.339	0.368
(Intercept)	−15.708	76.162	0.837
N	6693		

Zero-inflated negative binomial regression analysis of *bari*-level acute lower respiratory infection (ALRI) mortality rates in children under 2 years of age in Matlab, Bangladesh, 1989–1996. Data source: icddr,b, Matlab Health and Demographic Surveillance System

^a^Centered to the mean and scaled by the standard deviation

**Fig. 3:**
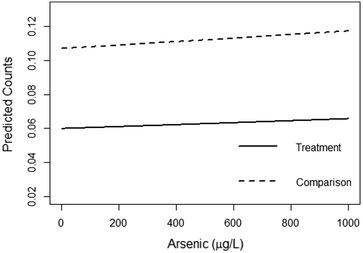
Predicted ALRI mortality in children under 2 years. The results of the zero-inflated negative binomial regression are used to examine the predicted mortality in Matlab *baris* under different scenarios of receiving treatment. Exposure to inorganic arsenic in their drinking water is varied from 0 to 1000 µg/L

The results of the ZINB model can be expressed as the expected mean counts for each *bari*. Figure 3 shows these counts predicted for varying levels of arsenic for a *bari* in the treatment and control areas while holding constant SES at low, and cost distance and person-years at their means. The expected count of ALRI deaths only slightly increases with increasing arsenic, but, at all levels of exposure, *baris* in the treatment area, on average, experience lower numbers of ALRI deaths than the comparison area. Table 3 shows the results of the ZINB models stratified by treatment and comparison areas in the upper and lower panels of the table, respectively. When analyzed separately, the patterns of associations remain largely consistent with the combined model. Higher socioeconomic status *baris* in both the treatment and comparison areas have lower ALRI mortality risk in young children. In the comparison area, one difference that emerges is that increasing cost distances are associated with lower counts of ALRI mortality.

**Table 3:** Regression results of stratified models

	β	SE	*p*
*Treatment area*			
Negative binomial model			
Arsenic (100 µg/L)	0.010	0.042	0.807
High socioeconomic status	−0.348	0.140	0.013
Cost distance to clinic^a^	0.125	0.147	0.396
(Intercept)	−4.777	0.195	0.000
Log(theta)	0.674	0.559	0.228
Zero-inflated model			
Arsenic (100 µg/L)	−2.921	2.742	0.287
High socioeconomic status	0.502	1.677	0.765
Cost distance to Clinic^1^	1.945	1.488	0.191
(Intercept)	−3.214	1.405	0.022
N	3251		
*Comparison area*			
Negative binomial model			
Arsenic (100 µg/L)	0.008	0.022	0.702
High socioeconomic status	−0.302	0.090	0.001
Cost distance to clinic^a^	−0.151	0.058	0.010
(Intercept)	−4.306	0.084	0.000
Log (theta)	2.807	1.815	0.122
Zero-inflated model			
Arsenic (100 µg/L)	−1.686	3.006	0.575
High Socioeconomic Status	−1.310	1.712	0.444
Cost Distance to Clinic^a^	−5.137	3.003	0.087
(Intercept)	−8.242	2.693	0.002
N	3442		

Zero-inflated negative binomial regression analysis of *bari*-level acute lower respiratory infection (ALRI) mortality rates in children under 2 years of age in Matlab, Bangladesh, 1989–1996 by treatment versus comparison area. Data source: icddr,b Matlab Health and Demographic Surveillance System

^a^Centered to the mean and scaled by the standard deviation

**Fig. 4:**
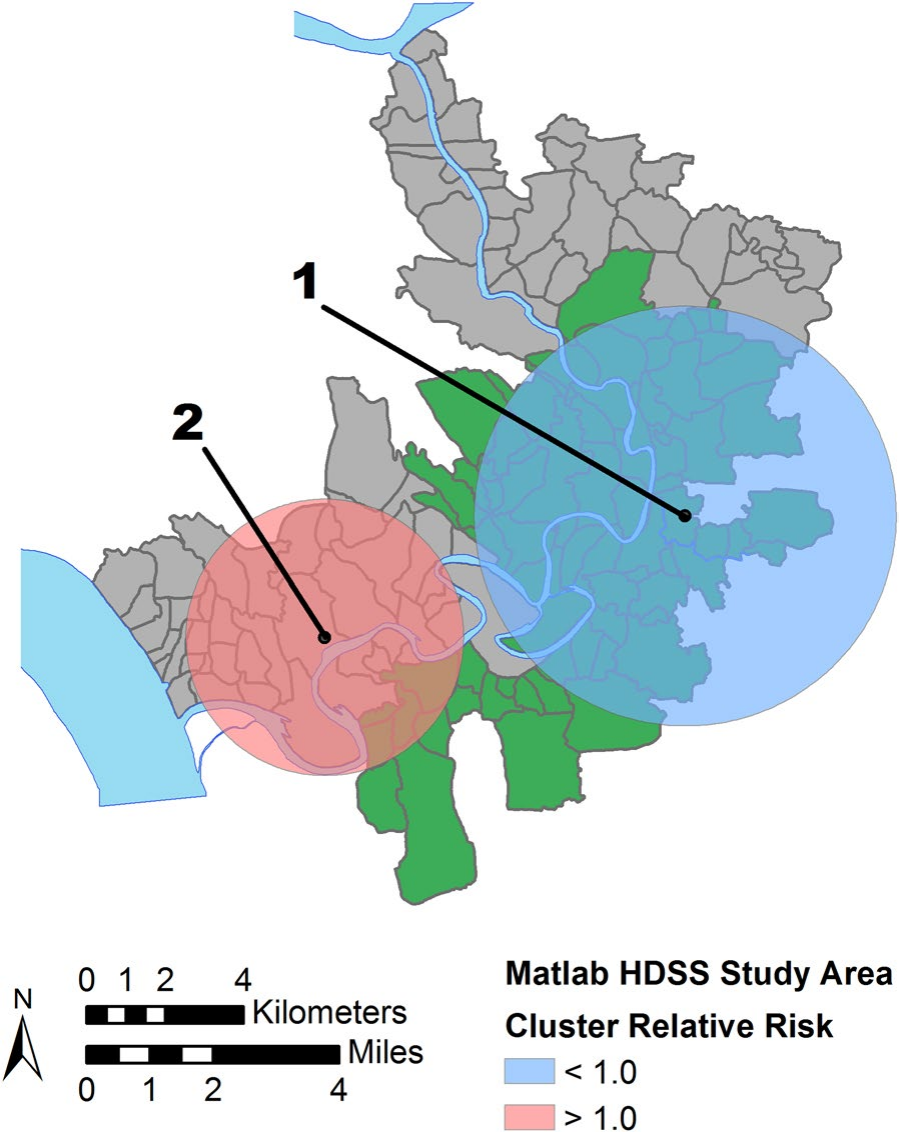
Results of an unadjusted spatial scan statistic. Statistically significant non-overlapping clusters of >1.0 (high, *red*) and <1.0 (low, *blue*) relative risk of mortality from acute lower respiratory infections to children <2 years (1989–1996) are found in treatment and comparison areas, respectively

**Fig. 5:**
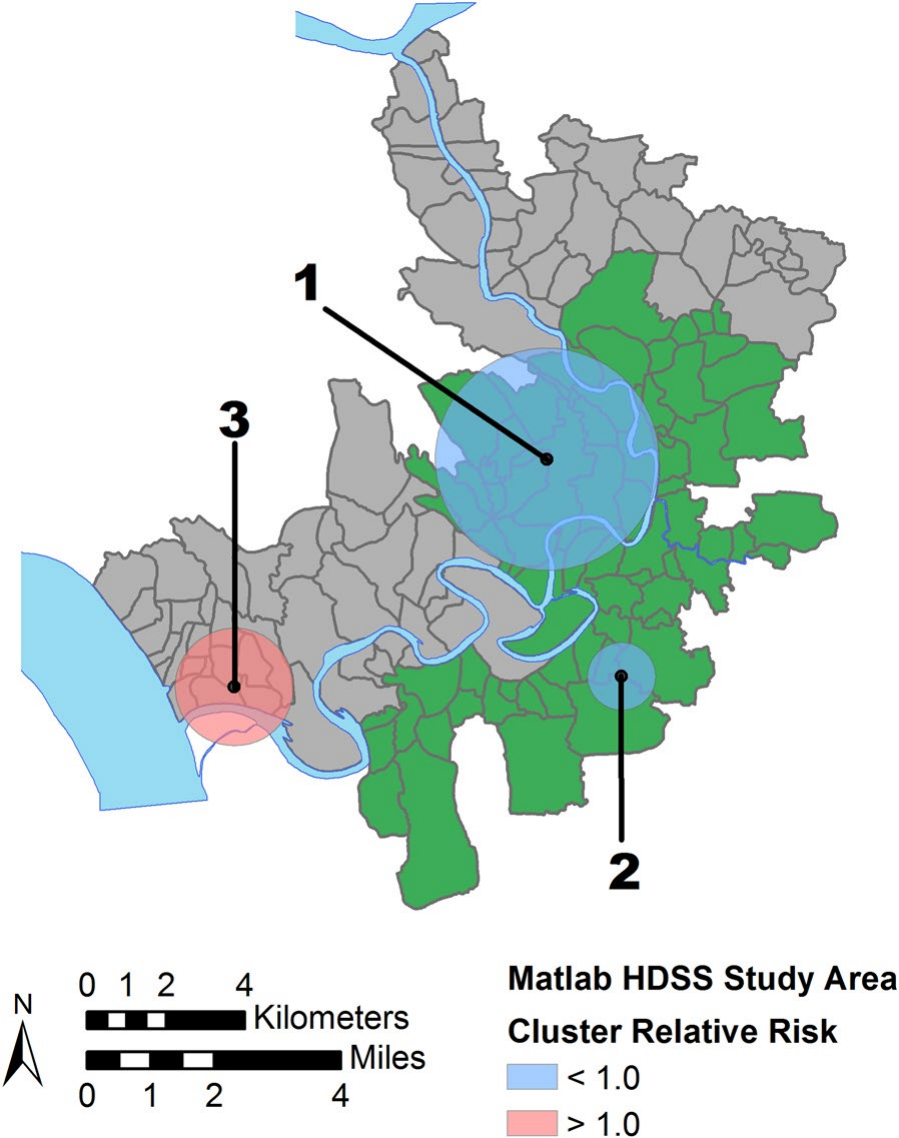
Results of an unadjusted spatial scan statistic limited to only reporting clusters containing less than 10 % of the population at risk. Clusters of ALRI mortality found after limiting the maximum reportable cluster size follow the same pattern as the standard, unadjusted model (see Fig. 4) with elevated risk in the comparison and lowered risk in the treatment area

**Fig. 6:**
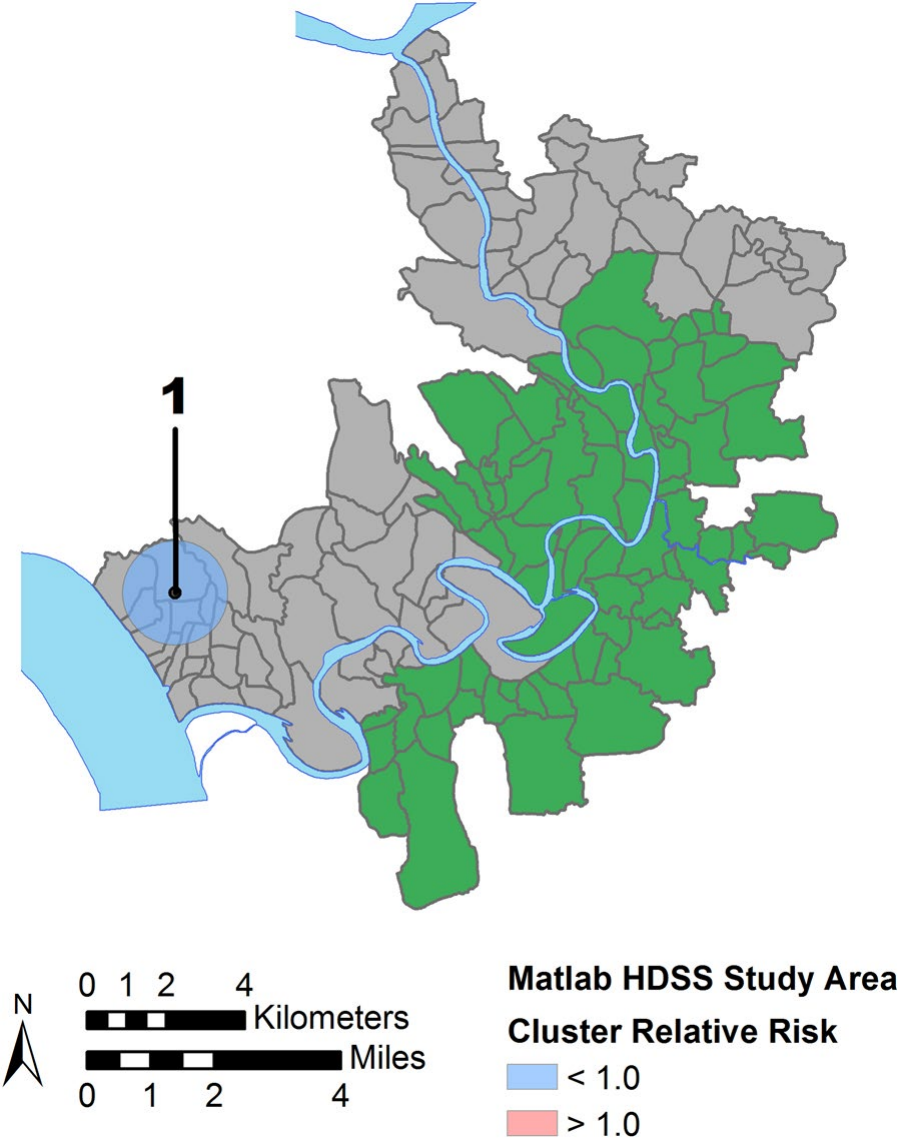
Results of a spatial scan statistic adjusted for the location of the treatment area. After adjusting for the location of treatment area villages (shown in *green*) only one low-risk statistically significant non-overlapping cluster of acute lower respiratory infection mortality is found in the southwestern portion of the study area

Figure 4 and related Table 4 show the results of the unadjusted scan statistic which identified two statistically significant clusters. The most likely cluster is a large area of lower mortality risk (relative risk, RR = 0.53, *p* = 0.000) centered over the treatment area. The second most likely cluster is an area of elevated risk (RR = 1.45, *p* = 0.051) in the southwest area of Matlab in the comparison area. The results of sensitivity testing of the unadjusted model, limiting the maximum population size to ≤10 % of the total, are shown in Fig. 5 and Table 4. While three statistically significant clusters are found, they all follow the already reported pattern of an area of low risk in the treatment area and an area of higher risk in the southern comparison area. Therefore, further spatial scan tests proceeded with the 50 % population limit. Figure 6 and Table 4 show the results after adjusting for the location of the treatment area. The most likely cluster is now an area of lower risk (RR = 0.41, *p* = 0.046) in the southwestern edge of the study area, part of the comparison area. There are no longer any areas of significantly elevated risk and the two clusters initially identified in the unadjusted analysis are no longer significant. The final spatial scan statistic test included adjustments using the combined ZINB model (e.g., treatment area, socioeconomic status, cost distance, arsenic exposure). After this adjustment the previously identified cluster of lower than expected ALRI mortality risk is no longer significant at the α = 0.1 level and no significant clusters are found in the study area (no map shown).

**Table 4:** Results of cluster analyses

	Observed deaths	Expected deaths	Radius (km)	Relative risk	Likelihood ratio	*p* value
Unadjusted (Fig. 4)						
Cluster 1	141	229.9	5.4	0.53	26.4	0.000
Cluster 2	259	197.8	3.5	1.45	11.8	0.051
Unadjusted, max size ≤10 % of population at risk (Fig. 5)						
Cluster 1	36	77.8	2.8	0.44	15.2	0.002
Cluster 2	0	14.3	0.9	0.00	14.4	0.003
Cluster 3	96	58.0	1.5	1.74	11.3	0.061
Treatment area adjusted (Fig. 6)						
Cluster 1	22	52.0	1.3	0.41	11.6	0.046
Model adjusted (*no figure*)						
	No statistically significant clusters found					

Spatial cluster analysis of childhood deaths from acute lower respiratory infections (ALRI) in Matlab, Bangladesh, 1989–1996. Results are from the spatial scan statistic implemented in SaTScan and include only statistically significant clusters at the α = 0.1 level

## Discussion

While previous studies of acute lower respiratory infections have primarily focused on individual-level risk factors [4, 6, 9], this research sought to highlight the broader contextual and environmental characteristics that can influence mortality rates. Part of the objectives of this study were to evaluate these risk factors for childhood ALRI mortality in the contexts of a community-based control program and exposure to inorganic arsenic from contaminated drinking water in Matlab, Bangladesh. This study found that living within the area served by the ALRI control program was strongly associated with reduced mortality rates measured at the *bari* level. This finding is consistent with previous studies [7, 13, 40] that found that the control program in Matlab was successful at reducing ALRI mortality in young children by up to 50 % during the period from 1989 to 1993. The study of ALRI mortality by Ali et al. [7] used a spatial filtering technique to map smoothed rates and qualitatively observed fewer areas of elevated rates in the Matlab MCH/FP treatment area. The present study also detected that pattern and the spatial scan statistic allows for statistical inferences which show that the spatial clustering is significant but that controlling for the treatment area and population distribution explains much of this local-scale variation.

In the present study, we also hypothesized that exposure to arsenic could be associated with increased ALRI mortality. Recent studies in Matlab and elsewhere have found that arsenic exposure is associated with increases in adverse pregnancy outcomes and infant mortality [65], as well as with harm to the lungs including coughs, cancer, and infections [29, 32–34]. Adding to the biological plausibility for the potential association with ALRI mortality are studies which have shown that arsenic’s toxic effects suppress the immune system, particularly in children [20], and damages cellular DNA and chemical receptors in lung tissue providing the opportunity for infections [21, 22]. However, in a zero-inflated negative binomial analysis at the *bari*-level used in this study, increased arsenic levels were not associated (*p* = 0.644) with ALRI mortality in children <2 years.

This study has extended the previous evaluation of the Matlab ALRI control program by Ali et al. [7] by including three additional years of mortality records as well as by incorporating measures of socioeconomic status and arsenic exposure that have not been previously used. *Baris* with higher SES were associated with lower rates of child mortality from ALRI in this study even after controlling for the treatment area effect. A case–control study on child ALRI mortality in Gambia did not find a significant association with SES [9], and, similarly, a prospective cohort study of children under 5 years in Matlab did not find an association with incidence of respiratory infections and various sociodemographic measures [6]. However, other studies have found various measures of social status including income, home ownership, and education to be predictive of respiratory infections in various age groups and country settings [53, 54, 66]. SES may affect respiratory infections by increasing exposure to pathogens in crowded living quarters or by decreasing an individual’s immune status due to stress or poor nutrition [58]. The conflicting findings in the literature may be due to differences in the specific measure of SES used in each study. Further work is needed to explore the role of SES in ALRI.

The cost distance to the nearest treatment center was also found to be associated with lower rates of ALRI death, though this finding was only statistically significant in the comparison area. Ali et al. [7] found that decreased access to care as measured by cost distance was associated with reduced mortality rates. While we expected that limiting access to care would increase mortality, this opposite finding is possible evidence of a reporting bias with more distant *baris* less likely to accurately report cases. Studies of diarrheal diseases in Matlab have found a similar pattern of decreased case numbers of diarrheal diseases with increasing distance from the hospital [50, 67].

Previous studies of mortality in Matlab using the spatial scan statistic have found visually similar patterns and areas of locally-clustered mortality related to the placement of the treatment area. In a village-level analysis of all-cause mortality in children under 5 years old between 1998 and 2002, Alam et al. [15] found significant clusters of elevated mortality centered on the northern and southern areas of Matlab as well as a secondary cluster of high risk along the eastern edge of the study area, after adjusting a space–time scan statistic for education and economic status. Using a spatial scan statistic on fetal and infant deaths between 1991 and 2000 and adjusting for age, parity, education, and SES, Sohel et al. [68] found a large cluster of significantly lower rates in central Matlab (coincident with the treatment area), and a smaller, but also significant cluster of elevated rates, in the southwestern portion of the comparison area. Visually these results are remarkably similar to those found in the unadjusted analysis of the present study. Sohel and colleagues [68] suggested that the clustering in fetal and infant mortality could be due to differences in arsenic exposure as they found significantly higher levels of arsenic in the wells used within the higher risk cluster. However, they did not adjust their scan statistic for arsenic level and test whether they could explain their observed spatial variation. Arsenic concentration was found to vary significantly between *baris* in the treatment and comparison areas (μ_treatment_ = 186.9, SD = 183.0 vs. μ_comparison_ = 253.1, SD = 225.3, *p* < 0.001), and a measure of arsenic exposure was incorporated into the final model-adjusted scan statistic; however, as the results of the ZINB model show, our estimate of potential arsenic exposure was not associated with ALRI mortality after adjusting for the differences in the population at risk, treatment area, SES, and cost distance. Therefore, we do not expect that arsenic alone would explain the clustering observed in the present study. The geographic pattern of the ALRI program implementation appears to be crucial for understanding the geographic patterns of child mortality in Matlab.

After adjusting for only the treatment area, which explained the two initial clusters, a new cluster of lower ALRI mortality emerged in the southwestern edge of the study area. This cluster of lower risk was unexpected, and it indicates an area of unexplained variation. The cluster could be the result of underreporting of ALRI deaths due to its location on the far edge of the study area, as discussed above. Another possibility is that these *baris* are receiving care elsewhere (e.g. outside of the Matlab study area) and so are not affected in the same way by the Matlab program placement. Ali et al. [7]. found that reduced ALRI mortality was associated with greater access to local allopathic doctors. Data on the locations of allopathic doctors and care-seeking behaviors were not available for this study. In the final spatial scan test, using the ZINB model to adjust the model, this cluster is no longer significant, indicating that the variation in expected counts has been explained by the addition of the other covariates and model form.

Strengths of this study include the detailed records collected in the demographic surveillance system in Matlab which enables accurate reporting of ALRI deaths and estimates of the population at risk. That these records can be linked in a geographic information system to spatial data on housing locations and tubewells with measured arsenic concentrations further enhances this study and allows for additional environmental and contextual variables to be considered. A limitation of this study is the arsenic exposure measure. The arsenic data come from 2002 to 2003 and are applied to the earlier study period of 1989–1996, which may bias the exposure estimate if arsenic varies over time. Evidence from several studies in Bangladesh, though limited, suggests that arsenic levels in wells are generally consistent over time [42, 51, 52]; however, it is not known which specific wells existed and were used by households during the study period. As arsenic contamination became more well-known in the 1990s, wells installed between 1996 and the arsenic survey in 2002 were likely deeper to access clean water while older wells which may have been broken or removed by 2002 (but were in use during the ALRI program) were typically shallower and, thus, more likely to be contaminated. These potential changes in wells over the years between our study period and arsenic survey would likely bias the arsenic exposure downward and averaging all tubewells belonging to a *bari* also potentially reduces the estimated exposure measure. These steps likely produce a conservative estimate of a *bari’s* true arsenic exposure and may explain the lack of a significant association between arsenic exposure and ALRI mortality in this study. Other environmental variables could be important to consider. For example we do not have a measure of indoor air pollution, yet this measure may be contributing to the observed SES association. Solid fuel use, which contributes to indoor air pollution and negative health effects, is more likely among poorer households in Matlab [69].

## Conclusion

This work provides one of the few examples of using regression models to adjust the spatial scan statistic and thereby incorporate continuous covariates and more complex models forms. Our initial results of adjusting the scan statistic for only treatment area, while explaining some of the larger clusters, produced an unexpected cluster of lower mortality risk. The model-adjusted scan statistic was able to explain this area of variation. Substantively, as acute lower respiratory infections continue to be a major cause of illness and death for children in Bangladesh and around the world, it is important to evaluate the effectiveness of community-based intervention strategies on population health. The results of this study indicate that child mortality form ALRI does cluster spatially in Matlab. However these patterns are largely explained by the placement of the treatment program. These results confirm the findings from previous non-spatial studies that the ALRI control program is effective at reducing mortality, though even in the presence of such an effective control program, household socioeconomic status may continue to influence the mortality patterns and this finding requires further study. More broadly, this study demonstrates the importance of geographic studies to highlight areas of significantly elevated or reduced mortality in order to evaluate and improve public health intervention programs.

## Abbreviations

AIC: Akaike Information Criterion
ALRI: acute lower respiratory infection
CHW: community health worker
HDSS: health and demographic surveillance system
HG-AAS: hydride generation atomic absorption spectrometry
icddr,b: formerly the International Centre for Diarrhoeal Disease Research, Bangladesh
MCH/FP: maternal and child health, and family planning program
MGIS: Matlab Geographic Information System
PCA: principal component analysis
RR: relative risk
SES: socioeconomic status
ZINB: zero-inflated negative binomial
